# A systematic review and meta-analysis of incidence trends and risk factors for metachronous gastric lesions following endoscopic resection

**DOI:** 10.1080/07853890.2025.2521443

**Published:** 2025-06-25

**Authors:** Meixi Gong, Xuanbing Li, Ruijun Xin, Yuansen Ma, Xiaomei Wu, Yan Zhao, Yu Sun, Bo Zhu

**Affiliations:** a Department of Cancer Prevention and Treatment, Cancer Hospital of China Medical University, Liaoning Cancer Hospital & Institute,Shenyang, China; b Department of Public Health, Dalian Medical University, Dalian, Liaoning, China; c Department of Clinical Epidemiology and Center of Evidence-Based Medicine, The First Hospital of China Medical University, Shenyang, China; d Department of Gastric Surgery, Cancer Hospital of China Medical University, Liaoning Cancer Hospital & Institute, Shenyang, China; e Liaoning Provincial Health Service Center, Shenyang, Liaoning Province, China

**Keywords:** Metachronous gastric lesions, early gastric cancer, risk factors, meta-analysis

## Abstract

**Background and Aims:**

Metachronous gastric lesions (MGLs) are a significant concern after endoscopic resection (ER) for precancerous lesions and early gastric cancers (EGCs). The follow-up strategy for the development of MGLs after ER remains unclear. The aim of this study was to compare the trend in the cumulative MGL incidence after ER and to identify risk factors of MGLs.

**Methods:**

We searched four databases (PubMed, Embase, Web of Science Core Collection and Cochrane Library) for studies reporting on patients who underwent ER, and performed meta-analysis. A dose-response analysis was performed to examine the association between follow-up time and cumulative MGL incidence after ER.

**Results:**

Of the 423 studies initially retrieved, 18 studies were finally included in the meta-analysis. The overall cumulative MGL incidence after ER was 9.4% (95% confidence interval (CI) 7.2%−1.9%). The cumulative MGL incidence in China (5.4%, 95% CI: 4.1%−6.8%) was significantly lower than those in Korea (10.2%, 95% CI: 7.2%–13.5%) and Japan (9.0%, 95% CI: 7.0%–13.2%). Dose-response analysis showed that cumulative MGL incidence increased rapidly during 3–7 years follow-up after ER. Male gender (HR = 1.283, 95% CI: 1.029–1.601), older age (HR = 1.025, 95% CI: 1.010–1.040), *Helicobacter pylori* (*H. pylori*) infection (HR = 1.573, 95% CI: 1.048–2.362), severe intestinal metaplasia (IM) (HR = 3.423, 95% CI: 1.830–6.042) were significantly associated with MGLs after ER.

**Conclusions:**

Nearly 10% of patients develop MGLs after ER. Older age, persistent *H. pylori* infection, severe intestinal metaplasia, and male sex were independent risk factors. Given the increased incidence 3 year after ER, surveillance with intensified follow-up is necessary for high-risk patients.

## 1. Introduction

According to GLOBOCAN 2022, gastric cancer remains one of the fifth common malignancies worldwide in terms of both incidence and mortality rates, with the highest incidence in East Asia [1]. Since the implementation of the National Cancer Screening Program (NCSP) in South Korea, the detection rate of early gastric cancer (EGC) has significantly increased rising from 39% in 2001 to 73% in 2016; in Japan, the rate of early detection of gastric cancer has exceeded 70% [2–4]. The improvement of national health awareness and the establishment of screening programs have led to a rise in the early detection and diagnosis of EGC [3,5].

In terms of treatment, endoscopic resection (ER), especially endoscopic submucosal dissection (ESD), has become the standard treatment for EGC, which has gradually replaced traditional surgery due to its advantages of minimally invasiveness, safety and preservation of organ function [6–8]. In East Asian countries with a high incidence of gastric cancer (male ASR: 23.0/100,000; female ASR:9.7/100,000), national guidelines have designated ESD as the preferred treatment for EGC and precancerous lesions [9–11]. Notably, even in low-incidence regions such as Europe, ESD is now recommended as first-line therapy for most superficial gastric lesions [12]. However, metachronous gastric lesions (MGLs) in the remaining gastric mucosa remain a significant challenge.

The MGL incidence exhibits significant variations across different regions and populations. In South Korea, the MGL incidence was 13.8% over 29 months [13]. In Japan, the MGL incidence reached 9.5%, 13.1% and 22.7% at 5, 7 and 10 years, respectively [14]. The MGL incidence in China demonstrated a relatively moderate progression, with rates of 3.5%, 5.1% and 6.9% observed at 3-, 5- and 7-year follow-up, respectively [15].

A study in Japan included 7,242 patients who had ER. After 5 years, six out of seven people who died from stomach cancer-related problems had cancer caused by MGLs [16]. Meanwhile, Huang’s study found a difference in the peak incidence of metachronous gastric cancer (MGC) of EGC and high-grade dysplasia (HGD), MGC happened most often 4 to 5 years after HGD resection, but 2 to 3 years after EGC resection [17] These results underscored the influence of MGLs on long-term outcomes.

Therefore, we conducted a systematic review and meta-analysis to evaluate the incidence trend and potential risk factors of MGLs after ER. We also used a dose-response model to evaluate the quantitative relationship between follow-up time and the MGL incidence, providing evidence-based evidence for optimizing individualized follow-up regimens.

## 2. Methods

We conducted a systematic review and meta-analysis based on the guidelines outlined in PRISMA [18]. Our research was registered on the Open Science Framework (10.17605/OSF.IO/CDV23).

### 2.1. Search strategy

A comprehensive literature search was conducted in the PubMed, Embase, Web of Science Core Collection and Cochrane Library databases from the establishment of the database to 31 March 2025. The search terms ‘gastric’, ‘endoscopic resection’, and ‘metachronous’ (pertaining to multiple lesions and second primary tumors) were used to construct the search strategy in all databases. The results were exported and deduplicated in EndNote X9 using Bramer’s method [19]. The search strategies for all the databases are presented in Table S1.

### 2.2. Definition

The term ‘MGL’ is used to describe new lesions that are detected more than 1 year after ER, near the primary lesion [20]. The term ‘EGC’ refers to cancerous tissues confined to the mucosal and submucosal layers, with or without regional lymph node metastasis. Gastric precancerous lesions are defined as pathological changes that have been shown to be closely associated with the development of gastric carcinoma. These changes are classified according to the severity of the lesions, with the classification system differentiating between low-grade intraepithelial neoplasia/dysplasia (LGIN/LGD) and high-grade intraepithelial neoplasia/dysplasia (HGIN/HGD) [21].

### 2.3. Study selection and inclusion and exclusion criteria

Two investigators (MG and BL) screened titles and abstracts for eligibility and reviewed the full text of all designated articles. Any disagreements regarding study inclusion were resolved through discussion and consensus, with a third investigator (BZ) available to mediate if necessary. Studies that met the inclusion criteria were selected for data extraction.

Inclusion criteria were: (1) Patients: Patients diagnosed with EGC or precancerous lesions (LGD or HGD) who underwent ER (include ESD or EMR) and had a follow-up time more than 1 year, (2) Intervention: Presence of at least one factor for MGLs, (3) Comparison: Absence of the aforementioned influencing factors, (4) Reported the MGL incidence, (5) The primary outcome was the detection of MGLs after 1 year follow-up. The secondary outcome aimed to identify the risk factors associated with MGLs, as well as the characteristics of MGLs, (6) Randomized controlled trials (RCTs) or cohort studies.

The exclusion criteria were: (1) Studies with duplicated data (the study with the most comprehensive data was selected), (2) Case reports, reviews and commentaries, (3) Studies that dealt only with resection of EGC or precancerous lesions, (4) Studies with unclear definitions of metachronous lesions, (5) Studies with less than 1-year follow-up, (6) Studies with no outcome of interest.

### 2.4. Data extraction and risk of bias Assessment

Two investigators (RX and YM) extracted the relevant data from studies meeting inclusion criteria. The data extraction form included the following items: (1) Basic information about the study (first author, year of publication, study design), (2) Characteristics of the study population (sample size, study period, country, age, sex, smoking status, alcohol consumption, family history, initial multiplicity of lesions, tumour size, gastric mucosal atrophy, intestinal metaplasia (IM), *Helicobacter pylori (H. pylori)* status, serology-based diagnosis of atrophy, gross type, location, depth of invasion, histologic type, etc.), (3) Measurements reported for outcomes, statistical method and cumulative MGL incidence, (4) Follow-up situation and (5) Evaluation of the quality of the study. The raw data from each study in the data extraction table will be recorded. For risk factors such as age, size, location and endoscopic mucosal atrophy, more than two categories were typically reported, and where possible, data were regrouped for meta-analysis. For example, lesion morphology was grouped into elevated and flat/depressed lesions. Based on the Kimura-Takemoto classification, mild and moderate atrophy of endoscopic mucosal atrophy were combined and contrasted with severe atrophy [22].

The risk of bias was assessed by two independent investigators (MG and BL). Discrepancies were resolved through consensus meetings with a third investigator (BZ). Cochrane Risk of Bias Assessment Tool was used to evaluate RCTs [23]. This tool comprised seven criteria, each designed to evaluate and categorize the quality of a study as having low risk, unclear risk, or high risk of bias. Cohort studies were evaluated using the Newcastle-Ottawa Scale (NOS). The NOS comprised eight items distributed across three domains: selection, comparability and outcome. High-quality studies scored 7–9, meeting most criteria across all domains; moderate-quality studies scored 4–6, meeting some criteria but with flaws; low-quality studies scored 0–3, failing to meet several criteria.

### 2.5. Data analysis

The pooled cumulative MGL incidence across all included studies was calculated using a random-effects model, with the DerSimonian and Laird method. The results are presented in the form of forest plots. A random-effects model was used when significant heterogeneity was detected, as it provides a more conservative and generalizable estimate by accounting for both within- and between-study variability [24]. To assess the robustness of our findings, we conducted sensitivity analyses by sequentially excluding each study and re-running the meta-analysis [25]. Univariate and multivariate meta-regressions were conducted, with the multivariate meta-regression model employing a multi-model inference approach to select variables based on the minimum Akaike Information Criterion (AIC) value. Subgroup analysis and meta-regression were performed to measure combined estimates according to study characteristics (different countries, sample sizes (<500, ≥500), type of study (cohort study, RCT), year of publication (before 2019, after 2019), median follow-up time (≥3 years, <3 years) and gender) in order to assess potential heterogeneity. When there was no overlap in the 95% CI coverage between the two subgroups, it was also deemed to be a significant difference [26]. Previous research have indicated that the mean time to the development of MGLs after ER of EGC is 3.1 years [27].

A dose-response analysis between follow-up time and cumulative incidence was conducted using a one-stage robust error meta-regression (REMR) model, which applied inverse variance-weighted least squares regression and cluster-robust error variances. The MGL incidence was extracted for each time point in the survival curves of the included study, and this incidence was summarized for 1 to 10 years follow-up. Subgroup analysis was conducted according to the initial histologic type, the status of *H. pylori* eradication and different countries. The graphical survival data were extracted using the Digitizer tool in Origin 2022 by calibrating coordinates with two anchor points, automatically tracking data points, and validating consistency through temporal sorting [28].

The characteristics of MGLs were also analyzed. After the raw data were expressed as medians and quartiles, the mean ± SD was calculated. Following the methodology proposed by McGrath and Luo, the median values were converted to mean ± SD [29,30].

Hazard ratios (HR) and their corresponding 95% confidence intervals (CI) were extracted from the included studies for meta-analysis.

*P-*values were assessed using two-tailed tests, with statistical significance set at *p* < 0.05. All our statistical analyses were executed with the Stata software, version 17.0.

## 3. Results

### 3.1. Search strategy and study characteristics

**Figure 1.**
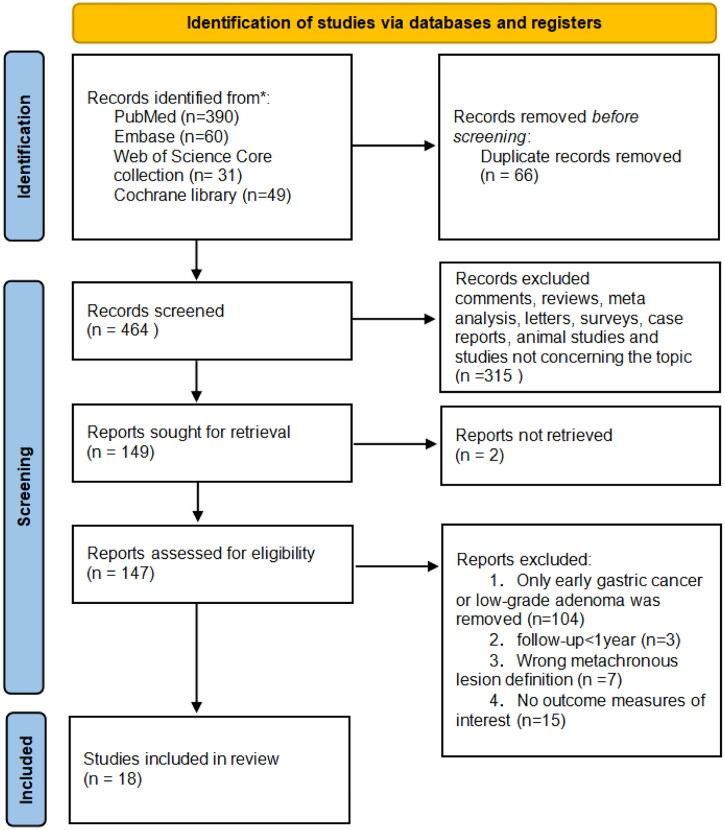
PRISMA flowchart.

Of the 423 studies retrieved, 123 studies were reviewed, and 18 studies, which contained 16 cohort studies and 2 RCTs, were finally included in Figure 1, Table S2 [15,17,31–46]. The comprehensive characteristics of the included studies are summarized in Table 1 and Table 2. The risk of bias assessment is presented in Figure S1 and Table S3.

**Table 1.** Basic characteristics of included studies.

First author, year	Country	Period	Patients	Events	Quality	Outcomes	Factors (HR)	Mean or median follow up
Tsunehiro Suzuki, 2024	Japan	2014.1–2018.12	369	27	High	Incidence	—	IQR 95 (54–127) months
Donghoon Kang, 2023	Korea	2015.1–2017.12	282	57	High	Incidence risk factor	Age, sex, location, HP	>5 years
Younghee Choe, 2023	Korea	2012.6–2022.7	677	87	High	Incidence	—	20 (12–91) months 24 (12–72) months
Keting Huang, 2023	China	2011.3–2018.3	286	12	High	Incidence risk factor	Age, sex, smoke, drink, family history, initial multiple, atrophy, IM, HP, histology type	median follow up 36 (12–105) months
Sunah Suk, 2023	Korea	2010.1–2020.12	190	26	High	Incidence risk factor	Sex, smoke, drink, PG *I* ≤ 70, PG I II ≤ 3, location, HP	median follow up 2.88 (1.02–14.28) years
Su Jin Kim, 2022	Korea	2009.9–2014.8	521	79	High	Incidence	—	5.3 ± 1.6 years
Shan-Shan Xu, 2021	China	2006.11–2019.9	814	37	High	Incidence risk factor	initial multiple, atrophy, HP	40.5 (12–146) months
Gisela Brito-Gonçalves,2020	Portugal	2012.1–2017.12	230	17	High	Incidence	—	33 (12–83) months
Hyun Jik Lee, 2018	Korea	2004.6–2012.6	643	100	High	Incidence	—	45.27 ± 27.59 (12–148) months
Charles J Cho, 2017	Korea	1999.4–2011.12	2779	92	High	Incidence risk factor	Age, sex, initial multiple, gross type, location, invasion depth , histology type	42 (26–58) months
Goh Eun Chung, 2017	Korea	2003.10–2014.12	316	36	High	Incidence	—	median follow up 4.2 (1.0–11.1) years
Hyuk Yoon, 2016	Korea	2004.10–2013.7	257	19	High	Incidence risk factor	Age, sex, smoke, family history, initial multiple, PG *I* ≤ 70, PG I II ≤ 3, location, HP, histology type	52 months
Seung Bae Yoon, 2016	Korea	2005.1–2013.8	947	89	High	Incidence risk factor	Age, sex, initial multiple, HP	median follow up 2.3 (1.0–8.9) years
Joo Hyun Lim, 2015	Korea	2001.4–2011.2	971	41	High	Incidence	—	37.1 ± 19.3 (12–131) months
Seon Young Park, 2014	Korea	2008.3–2011.3	228	24	High	Incidence	—	748.8 ± 34.7 days
Tomoyuki Boda, 2014	Japan	2002.4–2010.5	357	39	High	Incidence risk factor	Age, sex, initial multiple, PG *I* ≤ 70, PG I II ≤ 3, gross type, location, invasion depth , histology type	52.6 (12.2–113.4) months
Ji Min Choi, 2018	Korea	2005.4–2011.2	877	54	Low risk	Incidence risk factor	Age, sex, atrophy, IM, invasion depth , HP, histology type	71.6 months 71.7 months
Il Ju Choi, 2018	Korea	2003.8–2013.3	396	41	Low risk	Incidence	—	5.9 (4.0–8.2) years

**Table 2.** Presence or absence of metachronous lesion characteristics.

First author	Patients	Group	Age, years	Female sex, n/N	Atrophy	IM	Hp infection	Lesion size, mm	Lesion location (U/M/L)	Gross type	Histology type	Invasion depth
Tsunehiro Suzuki 2024	369	Without/with metachronous	Median (IQR) 73 (66–78) 77 (72–81)	107/342 5/27	(no/closed/open) 10/29/303 0/0/27	279/342 27/27	213/102/27 16/2/9	Median (IQR) 15 (10–23) 12 (10–19)	78/120/144→ 88/133/148 10/13/4→11/2/14		Differentiated/undifferentiated 330/12 27/0	M/SM 305/37 25/2
Donghoon Kang 2023	282	No AIG/AIG										
Younghee Choe 2023	677	Annual/ Biannual surveillance										
Keting Huang 2023	286	Without/with metachronous	≥60 years 171/262 21/24	76/262 6/24	66/262 12/24	106/262 18/24	Negative/eradicated/persisted127/68/67 7/4/13	mean ± SD 12.7 ± 6.1	88/16/158 9/2/13	Elevated/flat/depressed 102/103/57 8/8/8	EGC/HGD 122/140 13/11	
Sunah Suk 2023	190	Without/with metachronous	63.6 ± 10.5 69.6 ± 8.4	46/164 4/26	(PG *I* ≤ 70 and PG I/II ≤ 3) 37.1% 33.3%	111/164 20/26	last follow-up negative/positive 130/34 21/5	≥1.5 cm 75/164 11/26	19/32/113 2/6/18			
Su Jin Kim 2022	521	Biannual/ Annual surveillance			Normal ∼ C2/C3 ∼ O1/O2 ∼ O3 3/13/16 2/10/8		Infection 22/32 12/20			Elevated/flat/depressed 9/6/17 6/4/10	HGD/adenocarcinoma 5/27 2/18	
Shan-Shan Xu 2021	814	Without/with metachronous	median (IQR) 60.0 (53.0–68.0) 63.0 (55.0–70.5) ≥65 268/777 16/37	185/777 7/37	Severe 487/777 31/37	606/777 32/37	Infection 318/777 , 22/37 Eradication 127/777, 4/37	Median (IQR) 1.1 (0.7–1.8) 1.5 (0.8–2.0) cm	258/176/343 7/9/21	Elevated/flat/depressed 565/88/124 32/2/3	Differentiated/undifferentiated/ dysplasia 267/34/476 11/3/23	M/SM 742/35 34/3
Gisela Brito-Gonçalves 2020	230	Without/with metachronous	>60 136/181 13/17	88/181 7/17			Baseline Positive 42/156 2/17 last follow-up positive 4/152 1/17	>10mm 155/177 14/17	47/40/94 2/2/13		dysplasia/EGC 88/93 10/7 Differentiated/undifferentiated 90/3 7/0	
Hyun Jik Lee 2018	643	Metachronous initial/metachronous	65.3 ± 9.1 66.0 ± 8.5					14.8 ± 11.1 14.8 ± 11.8 >20mm 125/499 23/100	28/154/317 5/33/62	Polypoid/flat elevated/depressed 34/360/105 2/79/19	Differentiated/undifferentiated/ dysplasia 267/26/236 36/4/40	M/SM 468/31 95/5
Charles J Cho 2017	2779	With/without metachronous	Mean ± SD 63.8 ± 8.7 62.6 ± 16.8	19/92 662/2687				Mean ± SD 2.3 ± 1.4 2.1 ± 1.3	8/14/74 203/502/2180	Elevated /depressed 58/38 1553/1332	Differentiated/undifferentiated 76/5 2223/159	M/SM 74/7 2146/236
Goh Eun Chung 2017	316	LGD/HGD/carcinoma 17/5/14	59.4 ± 9.1 60.3 ± 4.3 63.6 ± 10.2				Persisted 8/3/8 Eradicated 3/2/1 Negative 6/0//5	8.2 ± 4.6 12.5 ± 5.0 14.6 ± 9.3	1/0/0 7/2/3 9/3/12		LGD13/;2/7 HGD0/0/0 EGC4/3/7	
Hyuk Yoon 2016	257	Without/with metachronous	≥65 150/257 8/257	71/74 3/19	PG *I* ≤ 30 and PG I/II ≤2.0 Not severe/severe 38/234 6/19	Absent or mild Moderate or severe IM (antrum) 117/121 8/11 (body) 180/58 8/11	Never/past/current 27/6/145 3/3/13	>2cm 27/238 3/19	Upper middle/lower 57/181 6/13		LGD/HGD/EGC 105/22/111 8/3/8	
Seung Bae Yoon 2016	947	Metachronous (Adenoma/ EGC)	68(64–74) 66(59–72)	9/30 15/59				9(6–14) 10(5–16)	2/15/18 9/20/34		Cancer/adenoma 14/21 32/31	
Joo Hyun Lim 2015	971	Without/with metachronous	62.79 (9.631) 66.60 (8.166)	233/873 5/42	470/683 21/27	727/837 39/40	Positive /total 471/838 13/40	1.863(1.216) 2.027(1.079) cm	83/262/524 2/10/30		Carcinoma 704/873 33/42 Undifferentiated cancer 87/872 3/42	SM 75/872 3/42
Seon Young Park 2014	228	Without/with metachronous	63.9 ± 9.5 64.4 ± 6.4	47/158 7/24	Severe PG 42/158 6/24		Positive 50 11	18.4 ± 0.7 18.8 ± 2.3	5/55/98 3/11/10	Protruding/Flat/elevate/Flat/Flat pressed 6/106/11/35 1/17/1/5	LGD/HGD /Differentiated/undifferentiated 57/46/52/3 7/7/10/0	
Tomoyuki Boda 2014	357	Metachronous	70.3 (50–88)	4/39	Closed/open 1/38			11.1 (3–20)	10/12/17	Elevated /depres18/21		M/SM 34/5
Ji Min Choi 2018	877	Metachronous (hp Eradication group/control group)	60.6 ± 9.4 60.7 ± 6.5	3/18 8/36	With/without 3/15 9/27	With/without 0/18 4/32		2.0 ± 1.2 1.9 ± 1.1	0/2/16 1/8/27		EGC/HGD/LGD 12/0/6 23/3/10 Differentiated/undifferentiated 11/1 20/3	M/SM 12/0 20/3
Il Ju Choi 2018	396	Metachronous (hp Eradication group/control group)	66.0 ± 5.3 60.7 ± 7.7	3/14 3/27				1.2 ± 0.6 1.7 ± 1.1	1/3/10 1/8/18		Differentiated/undifferentiated 14/0/0 25/1/1	M/SM 13/0/1 22/4/1

### 3.2. The cumulative incidence of MGL after ER

**Figure 2.**
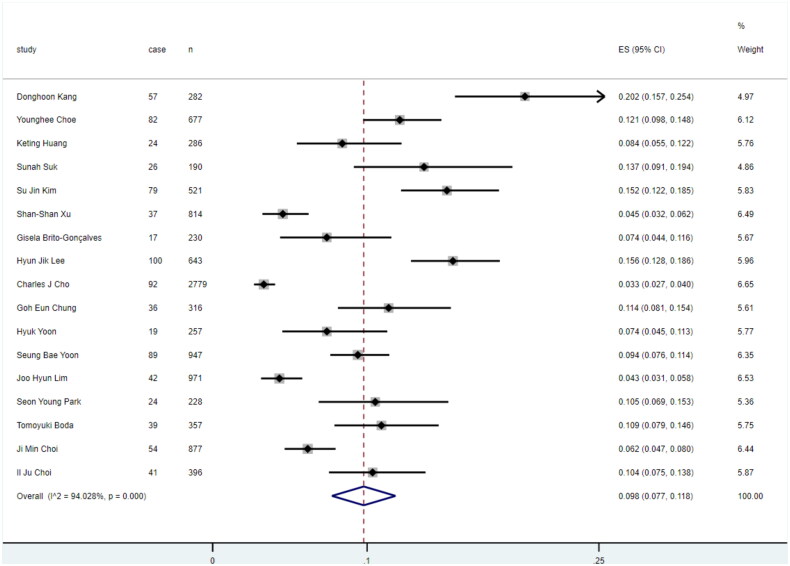
The cumulative metachronous lesions incidence.

We pooled data from 18 studies, resulting in an overall cumulative incidence of 9.4% (95% CI: 7.2%−11.9%, Figure 2). Sensitivity analysis demonstrated that no single study disproportionately influenced the pooled incidence estimate (Figure S6).

Subgroup analysis conducted based on these factors did not account for a significant portion of the between-study heterogeneity. However, we found that the MGL incidence of in China (5.4%, 95% CI: 4.1%−6.8%) was significantly lower compared to Korea (10.2%, 95% CI: 7.2%–13.5%) and Japan (9.0%, 95% CI: 7.0%–13.2%) (Figure 5). Meta-regression indicated that all p-values exceeding 0.05. This suggests that the examined covariates may not be primary drivers of the observed heterogeneity (Table 3).

**Table 3.** Meta-regression results.

Variable		Single	*P*	Multivariate	*P*
Country	Korea	0.96 (0.85–1.09)	0.582	0.96 (0.93–1.00)	0.054
	Japan	Ref		/	/
	China	1.00 (0.91–1.10)	0.925	/	/
	Portugal	0.98 (0.99–1.20)	0.844	/	/
Sample size	<500	Ref		1.03 (0.99–1.09)	0.145
	≥500	0.97 (0.92–1.02)	0.277	/	/
Publication year	Before 2019	Ref		0.96 (0.89–1.00)	0.061
	After 2019	1.02 (0.97–1.08)	0.292	/	/
Study type	Cohort study	1.01 (0/93–1.09)	0.716	/	/
	RCT	Ref		/	/
Follow up	≥3 years	Ref		/	/
	<3 years	1.02 (0.96–1.08)	0.470	/	/

### 3.3. Dose-response analysis between follow-up time and cumulative MGL incidence

**Figure 3.**
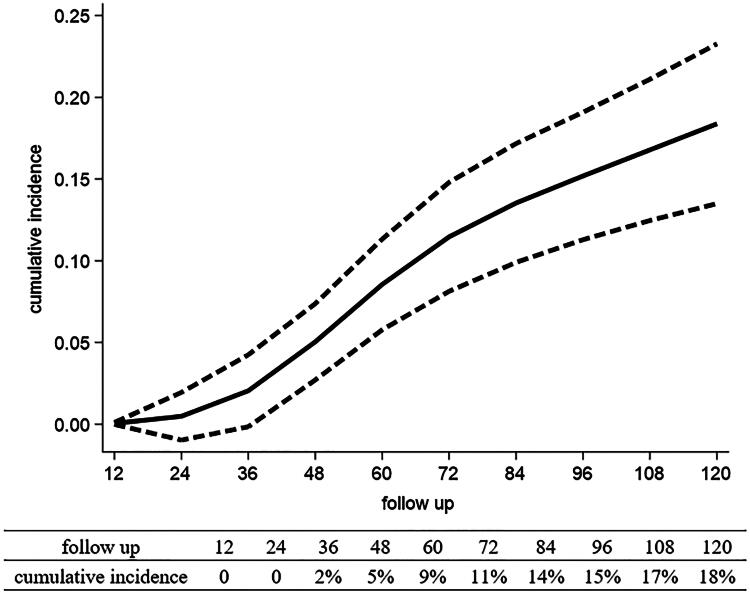
Dose-response analysis between follow-up time and cumulative metachronous lesions incidence.

Eleven studies were included in the dose-response analysis, revealing a relationship between follow-up time and cumulative MGL incidence. Within the first 3 years of follow-up, the cumulative incidence rate increased relatively steadily to 2% (95% CI: 0%–4%). However, a notable acceleration in cumulative incidence was observed between years 3 and 7, increasing from 2% (95% CI: 0%–4%) to 14% (95% CI: 10%–17%). After 7 years, the incidence rate slowed down, reaching 18% (95% CI: 13%–23%) at 10 years (Figure 3). Figures S2, S3 and S4 illustrate the results of subgroup analyses conducted based on histological type, *H. pylori* status and different countries.

### 3.4. Characteristics of MGLs after ER

The primary histology from 206 metachronous precancerous lesions and 194 metachronous EGCs were analyzed. Among the metachronous EGCs, the primary lesions were precancerous lesions in 67 patients (34%) and EGC in 127 patients (66%). Histology of the primary lesions and subsequent MGLs were presented in Table 4.

**Table 4.** Clinicopathological characteristics of metachronous gastric lesions.

Histology of primary lesion	Histology of metachronous lesion	n/N (%)
Precancerous lesions	Precancerous lesions	96/2543 (4.1)
	EGC	67/2829 (2.3)
EGC	Precancerous lesions	110/3144 (3.5)
	EGC	127/3430 (3.8)

Table 5 presents the detailed characteristics of MGLs after ER. The majority of MGLs exhibited flat or depressed morphology (208/289, 72.0%) and were predominantly located in the lower part of the gastric longitudinal axis (228/391, 58.3%) and 177 lesions were found in the same longitudinal location as the initial lesions (Table 5).

**Table 5.** Clinicopathological characteristics of metachronous gastric lesions.

		Longitudinal location				Cross-sectional location				Gross type				Histologic Type						Invasion depth	
First author, year	MGL	Same	Upper	Middle	Low	GC	LC	AW	PW	Same	Elevated	Flat	Depressed	Same	LGD	HGD	EGC	WD	UD	M	SM
Keting Huang, 2023	22	12	9	1	12	3	13		6	11	2	0	0			13		7	2	19	3
Su Jin Kim, 2022	52										15	10	27	9	4	8	5				
Gisela Brito-Gonçalves, 2019	17	11	2	2	13																
Hyun Jik Lee, 2018	100	56	7	37	56	25	29	19	27	69	8	71	21	56			37				
Charles J Cho, 2017	96	49	10	18	68						48		48			17		61	4	5	8
Hyuk Yoon, 2016	19		4	5	10										10	2	6				
Seung Bae Yoon, 2016	98	49	11	35	52												46				
Tomoyuki Boda, 2014	39		10	12	17						18		21							34	5

MGL: metachronous gastric lesions, GC: Greater curvature, LC: Lesser curvature, AW: Anterior wall, PW: Posterior wall, WD: Well differentiated, UD: Undifferentiated.

### 3.5. Risk factors of MGLs after ER

**Figure 4.**
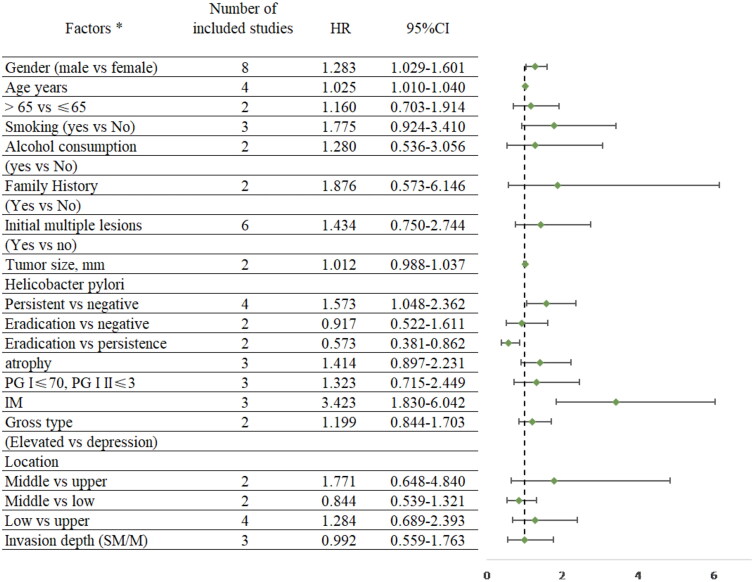
Factors of metachronous gastric lesions.

**Figure 5.**
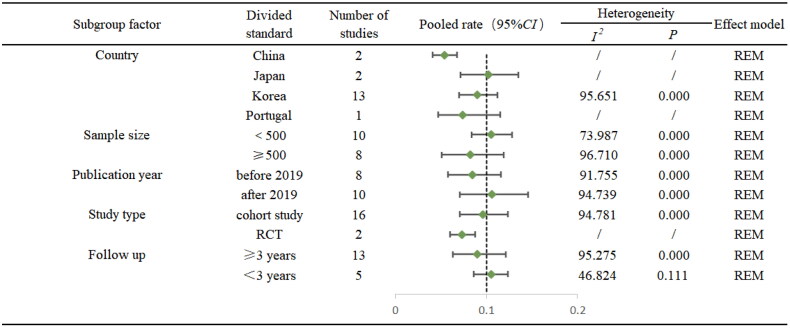
Forest plot of subgroup analysis.

Figure 4 summarized the detailed results on risk factors of MGLs after ER. Older age (HR = 1.025, 95% CI: 1.010–1.040), male gender (HR = 1.283, 95% CI: 1.029–1.601), *H. pylori* infection (HR = 1.573, 95% CI: 1.048–2.362) and severe IM (HR = 3.423, 95% CI: 1.830–6.042) were significant risk factors for the occurrence of MGLs. Smoking, alcohol consumption, family history and initial multiple lesions, gastric mucosal atrophy, tumour size, lesion morphology, location and depth of invasion were not significantly associated with the occurrence of MGLs, Figure S5 showed the details (Figure 5).

## 4. Discussion

Though patients with EGC and gastric precancerous lesions after ER are at risk for MGLs, follow-up strategy is unclear. Our study performed the dose-response relationship between follow-up time and cumulative MGL incidence, explored the characteristics and risk factors of MGLs in patients with EGC and gastric precancerous lesions after ER. We aimed to provide a basis for developing scientific follow-up strategies and surveillance guidelines to reduce MGLs risk and improve patient quality of life.

In contrast to the previous study that used the median follow-up time to predict cumulative MGL incidence after ER [47], we extracted the incidence data directly from the survival curves of each study and performed a dose-response analysis. Our analysis revealed a more precise depiction of the relationship between cumulative MGL incidence and follow-up time, characterized by the S-shaped curve. During the initial 3 years, the cumulative MGL incidence increased gradually. A sharp increase in cumulative MGL incidence was observed from 3 to 7 years; the upward trend then slowed. Based on the available evidence, 3 years after ER is a critical time point marked by a sharp rise in cumulative MGL incidence. Consequently, a uniform follow-up strategy may be inadequate. Considering the high-risk period between 3 and 7 years after ER, intensified surveillance should be implemented, particularly for individuals with identified risk factors.

This pattern likely reflects the influence of multiple factors on MGLs occurrence. Regarding histologic types, we observed a linear increase in EGC (Figure S3). However, the trend for precancerous lesions was similar to the overall cumulative MGL incidence pattern, a possible explanation could be that precancerous lesions might be overlooked during endoscopy due to their insidious location or atypical presentation, resulting in a delay in diagnosis [48]. Additionally, immune senescence and subsequent immune dysfunction are recognized as significant risk factors for cancer development, including the MGLs progression [49,50].

The significantly lower MGL incidence in China (5.4%) compared to those in Japan (9.0%) and South Korea (10.2%) may be attributed to the following factors: First, the detection of MGLs after ER in China is constrained by domestic healthcare realities. China’s gastric cancer screening is limited to selected high-risk regions [51], Japan and South Korea have established nationwide screening programs [52,53]. Systematic improvements in endoscopic equipment and standardized physician training in these countries have significantly enhanced MGLs detection accuracy during follow-up after ER [54,55]. In contrast, China faces challenges including insufficient endoscopic expertise and outdated equipment, resulting in higher rates of missed diagnoses. Furthermore, low-income populations exhibit reduced health awareness and financial barriers, contributing to low compliance with follow-up after ER and further limiting MGLs detection [51,53]. Second, patients with hereditary gastric cancer exhibit a higher CDH1 mutation rate in Japan, whereas this genetic variant occurs significantly less frequently in China [56,57]. Such inherited divergence may partially account for the disparity in MGL incidence across different population. Third, the high intake of traditional high-salt and fermented foods (notably pickled vegetables and fermented fish) in Japan and South Korea is significantly associated with gastric cancer risk [58,59]. In contrast, the lower consumption of such dietary patterns in China may serve as a contributing factor to the lower MGL incidence.

Our study found that the probability of patients with EGCs and precancerous lesions developing metachronous EGCs or precancerous lesions after ER ranged from 4.0% to 6.3%, highlighting the potential risk faced by these patients. Given the complexity and variability of disease progression, we further performed a comprehensive analysis of risk factors.

Both gender and age are risk factors for MGLs development. Males have a higher predisposition than females, and older individuals face increased risk due to prolonged exposure to carcinogens and declining immune function [60–63].

Our findings indicate a 42.7% reduction in MGL incidence after timely *H. pylori* eradication after ER. This protective effect aligns with previous studies by Choi and Khan, which reported approximately a 50% risk reduction associated with *H. pylori* eradication therapy and the degree of gastric atrophy has improved compared to the baseline [45,64]. In our findings, the cumulative MGL incidence is twice as high in patients without *H. pylori* eradication at 3 years after ER. MGLs are more prevalent in patients who have not eradicated *H. pylori*. The International Agency for Research on Cancer classified *H. pylori* as a Group I carcinogen in 1994 [20], emphasizing its role in the development of gastric cancer. Persistent *H. pylori* infection triggers a cascade of pathological changes, progressing from inflammation to atrophic gastritis, IM, dysplasia or intraepithelial neoplasia and potentially culminating in gastric cancer [65]. Given the pathogenic sequence, eradicating *H. pylori* is crucial to preventing the progression to more severe gastric lesions.

Additionally, we further found no significant difference in MGLs occurrence between patients who successfully eradicated *H. pylori* and those who were initially *H. pylori* negative, aligning with previous meta-analyses [66,67], which confirms that *H. pylori* eradication therapy can achieve similar protective effects to the *H. pylori* negative state, significantly reducing the risk of gastric cancer and MGLs. Therefore, immediate *H. pylori* eradication treatment for EGC or precancerous lesion patients diagnosed with *H. pylori* infection not only aids in the treatment of the current disease but also provides long-term health benefits.

In exploring the impact of endoscopic and histological factors, severe IM serves as a significant sign indicating the imminent occurrence of MGLs in the group after ER. Patients with severe IM had a 3.423-fold higher risk of MGLs compared to those with mild to moderate IM. Our finding aligns with a previous meta-analysis that EGC patients with IM had a sevenfold higher risk of MGC than those without IM [68]. Despite subtle differences among the study populations, we all further strengthened that IM serves as a significant predictor for the occurrence of MGLs after ER.

Our study has several strengths. First, in the selection of study subjects, we innovatively included cases with simultaneous resection of both precancerous lesions and EGCs, given their similar development pathways for MGLs. This approach enhances comprehensiveness and robustness compared to previous studies that focused on either condition individually. Secondly, to our knowledge, this is the first study to employ a dose-response analysis to illustrate the relationship between cumulative MGL incidence and follow-up time. The findings from this analysis offer clinicians a reliable foundation for devising individualized follow-up strategies, which can enhance the early detection, diagnosis and treatment of MGLs, ultimately improving patient outcomes and quality of life.

Our study has several limitations. First, based on the principle of non-transferability, the timing of *H. pylori* eradication can influence the development of MGLs. However, the original studies do not clearly specify this timing, potentially affecting our findings. Second, the number of studies from regions outside East Asia was limited, with only one study from Portugal. This geographical imbalance may introduce a degree of bias into the results. However, East Asia has a high incidence of gastric cancer, according to the Global Burden of Disease database. Lastly, the subgroup comparisons included in the dose-response analysis, particularly those related to the cumulative MGL incidence, lesion type and *H. pylori* status, were not adequately detailed in many of the original articles. Consequently, further research is essential to generate more robust estimates.

## 5. Conclusion

In conclusion, approximately 10% of patients develop MGLs after ER. Older age, persistent *H. pylori* infection, severe intestinal metaplasia, and male sex are independent risk factors. The cumulative incidence rises notably at 3 years after ER. Therefore, surveillance strategies should be risk-stratified, with intensive follow-up recommended for patients with risk factors, particularly beyond the third year. Our findings provide an evidence-based foundation for developing personalized surveillance protocols for high-risk populations and support the refinement of future clinical guidelines.

## Supplementary Material

Supplemental Material

## Data Availability

All data utilized in this study were extracted from previously published articles. The datasets supporting the findings are fully presented in the manuscript and its supplementary files. For access to further inquiries, please contact the corresponding author.
