# Supplementary material for: A systematic review and meta-analysis of incidence trends and risk factors for metachronous gastric lesions following endoscopic resection
Source: Ann Med. 2025 Jun 25;57(1):2521443. doi: 10.1080/07853890.2025.2521443 (PMC12931332; doi:10.1080/07853890.2025.2521443)
Supplement: Supplemental Material [file IANN_A_2521443_SM7117.zip › suppl_data/Supplementary Materials legend.docx]

**Supplementary Figures legend**

**Figure S1.** Quality assessment using Cochrane RoB2

**Figure S2.** Dose-response analysis of cumulative incidence versus follow-up time (China, Japan, Korea)

**Figure S3.** Dose-response analysis of cumulative incidence versus follow-up time (Early gastric cancer, precancerous lesions)

**Figure S4.** Dose-response analysis of cumulative incidence versus follow-up time (Eradication of Helicobacter pylori, non-eradication of Helicobacter pylori)

**Figure S5.** Forest Plots of risk factors of metachronous lesions (A Gender (male vs female), B Age years, C Age years (> 60 vs ≤60), D Smoking (yes vs No), E Alcohol consumption (yes vs No), F Family History (Yes vs No), G Initial multiple lesions (Yes vs no), H Tumor size, mm, I Helicobacter pylori (Persistent vs negative), J Helicobacter pylori (Eradication vs negative), K Helicobacter pylori (Eradication vs persistence), L Atrophy, M PG I≤70,PG I II≤3, N IM, O Gross type (Elevated vs depression), P Location (Middle vs upper), Q Location (Middle vs low),R Location (Low vs upper))

**Figure S6.** Sensitivity analysis of the incidence of metachronous lesions
