# Supplementary material for: A systematic review and meta-analysis of incidence trends and risk factors for metachronous gastric lesions following endoscopic resection
Source: Ann Med. 2025 Jun 25;57(1):2521443. doi: 10.1080/07853890.2025.2521443 (PMC12931332; doi:10.1080/07853890.2025.2521443)
Supplement: Supplemental Material [file IANN_A_2521443_SM7117.zip › suppl_data/Supplementary Table S2.docx]

**Table S2** The summary of detailed search strategies

| Database | Search strategy |
| --- | --- |
| PubMed | #1 gastric OR stomach  #2 ((endoscopic submucosal dissection) OR (ESD) OR (EMR) OR ("endoscopic mucosal resection") OR ("endoscopic resection"))  #3 metachronous OR "second cancer" OR "second primary" OR "multiple lesions"  #4 #1 AND #2 AND #3 |
| Embase | #4. #1 AND #2 AND #3 60  #3. 'endoscopic submucosal dissection'/exp OR 'endoscopic mucosal resection'/exp 18,495  #2. 'second primary neoplasm'/exp OR 'second cancer'/exp 22,799  #1. 'stomach cancer'/exp 169,887 |
| Web of Science | 1: TS=("gastric cancer")OR TS=(stomach cancer )OR TS=(gastric neoplasm) Editions: WOS.SCI,WOS.CCR Results: 120898  2: TS=("gastric cancer")OR TS=(stomach cancer )OR TS=(gastric neoplasm)AND TS=(dysplasia) Editions: WOS.SCI,WOS.CCR Results: 117539  3: ((((TS=("endoscopic resection")) OR TS=(endoscopic submucosal resection)) OR TS=(endoscopic submucosal dissection)) OR TS=(ESD)) AND TS=(EMR) Editions: WOS.SCI,WOS.CCR Results: 1762  4: (TS=(metachronous)) OR TS=(second primary cancer) Editions: WOS.SCI,WOS.CCR Results: 26359  5: #4 AND #3 AND #2 Editions: WOS.SCI,WOS.CCR Results: 31 |
| Cochrane Library | #1 MeSH descriptor: [Stomach Neoplasms] explode all trees 4142  #2 Gastric Cancer OR Familial Diffuse Gastric Cancers OR Stomach Cancer OR Cancers, Gastric OR Cancer of Stomach OR Stomach Cancers OR Cancer of the Stomach OR Gastric Cancer OR Cancer, Stomach OR Cancer, Gastric OR Cancers, Stomach OR Neoplasm, Gastric OR Gastric Neoplasm OR Stomach Neoplasm OR Neoplasms, Gastric OR Gastric Neoplasms OR Neoplasms, Stomach OR Neoplasm, Stomach 12623  #3 endoscopic submucosal dissection OR ESD OR EMR OR endoscopic mucosal resection OR "endoscopic resection" 3542  #4 metachronous OR "second cancer" OR "second primary" OR "multiple lesions" 2692  #5 #1 OR #2 12623  #6 #3 AND #4 AND #5 49 |
