# Supplementary material for: A systematic review and meta-analysis of incidence trends and risk factors for metachronous gastric lesions following endoscopic resection
Source: Ann Med. 2025 Jun 25;57(1):2521443. doi: 10.1080/07853890.2025.2521443 (PMC12931332; doi:10.1080/07853890.2025.2521443)
Supplement: Supplemental Material [file IANN_A_2521443_SM7117.zip › suppl_data/Supplementary Table S3.docx]

**Table S3** NOS scores for observational studies

| Study | Selection | | | | Comparability | | Outcome | | | Score |
| --- | --- | --- | --- | --- | --- | --- | --- | --- | --- | --- |
|  | 1 | 2 | 3 | 4 | 5A | 5B | 6 | 7 | 8 | Total |
| Tsunehiro Suzuki  2024 | ✔ | ✔ | ✔ | ✔ | ✔ | ✔ | ✔ | ✔ | ✔ | 9 |
| Donghoon Kang  2023 | ✔ | ✔ | ✔ | ✔ | ✔ | ✔ | ✔ | ✔ | ✔ | 9 |
| Younghee Choe  2023 | ✔ | ✔ | ✔ | ✔ | ✔ | ✔ | ✔ | ✔ |  | 8 |
| Keting Huang  2023 | ✔ | ✔ | ✔ |  | ✔ | ✔ | ✔ | ✔ | ✔ | 8 |
| Sunah Suk  2023 | ✔ | ✔ | ✔ | ✔ | ✔ | ✔ | ✔ | ✔ | ✔ | 8 |
| Su Jin Kim  2022 | ✔ | ✔ | ✔ | ✔ | ✔ | ✔ | ✔ | ✔ | ✔ | 9 |
| Shan-Shan Xu  2021 | ✔ | ✔ | ✔ | ✔ | ✔ | ✔ | ✔ | ✔ | ✔ | 9 |
| Gisela Brito-Gonçalves  2019 | ✔ | ✔ | ✔ | ✔ | ✔ | ✔ | ✔ | ✔ | ✔ | 9 |
| Hyun Jik Lee  2018 | ✔ | ✔ | ✔ | ✔ |  |  | ✔ | ✔ | ✔ | 7 |
| Charles J Cho  2017 | ✔ | ✔ | ✔ | ✔ | ✔ | ✔ | ✔ | ✔ |  | 8 |
| Goh Eun Chung  2017 | ✔ | ✔ | ✔ |  | ✔ | ✔ | ✔ | ✔ | ✔ | 8 |
| Hyuk Yoon  2016 | ✔ | ✔ | ✔ | ✔ | ✔ | ✔ | ✔ | ✔ | ✔ | 9 |
| Seung Bae Yoon  2016 | ✔ | ✔ | ✔ | ✔ | ✔ | ✔ | ✔ | ✔ | ✔ | 9 |
| Joo Hyun Lim  2015 | ✔ | ✔ | ✔ | ✔ | ✔ | ✔ | ✔ | ✔ | ✔ | 9 |
| Seon Young Park  2014 | ✔ | ✔ | ✔ | ✔ | ✔ | ✔ | ✔ | ✔ | ✔ | 9 |
| Tomoyuki Boda  2014 | ✔ | ✔ | ✔ | ✔ | ✔ | ✔ | ✔ | ✔ | ✔ | 9 |
