# Supplementary figures and images for: A systematic review and meta-analysis of incidence trends and risk factors for metachronous gastric lesions following endoscopic resection
Source: Ann Med. 2025 Jun 25;57(1):2521443. doi: 10.1080/07853890.2025.2521443 (PMC12931332; doi:10.1080/07853890.2025.2521443)

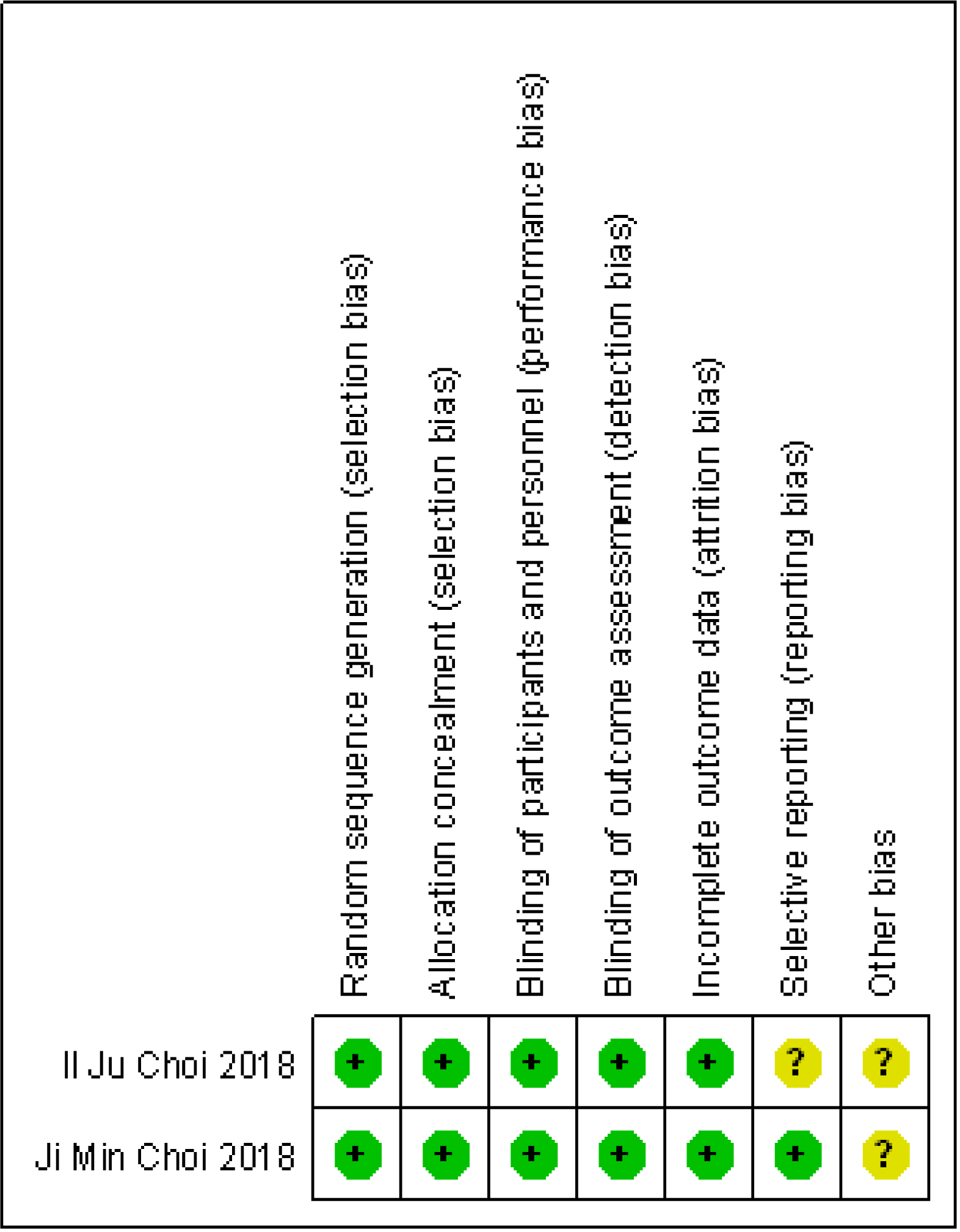

Supplement: Supplemental Material [file IANN_A_2521443_SM7117.zip › suppl_data/Supplementary Figure S1.tif]

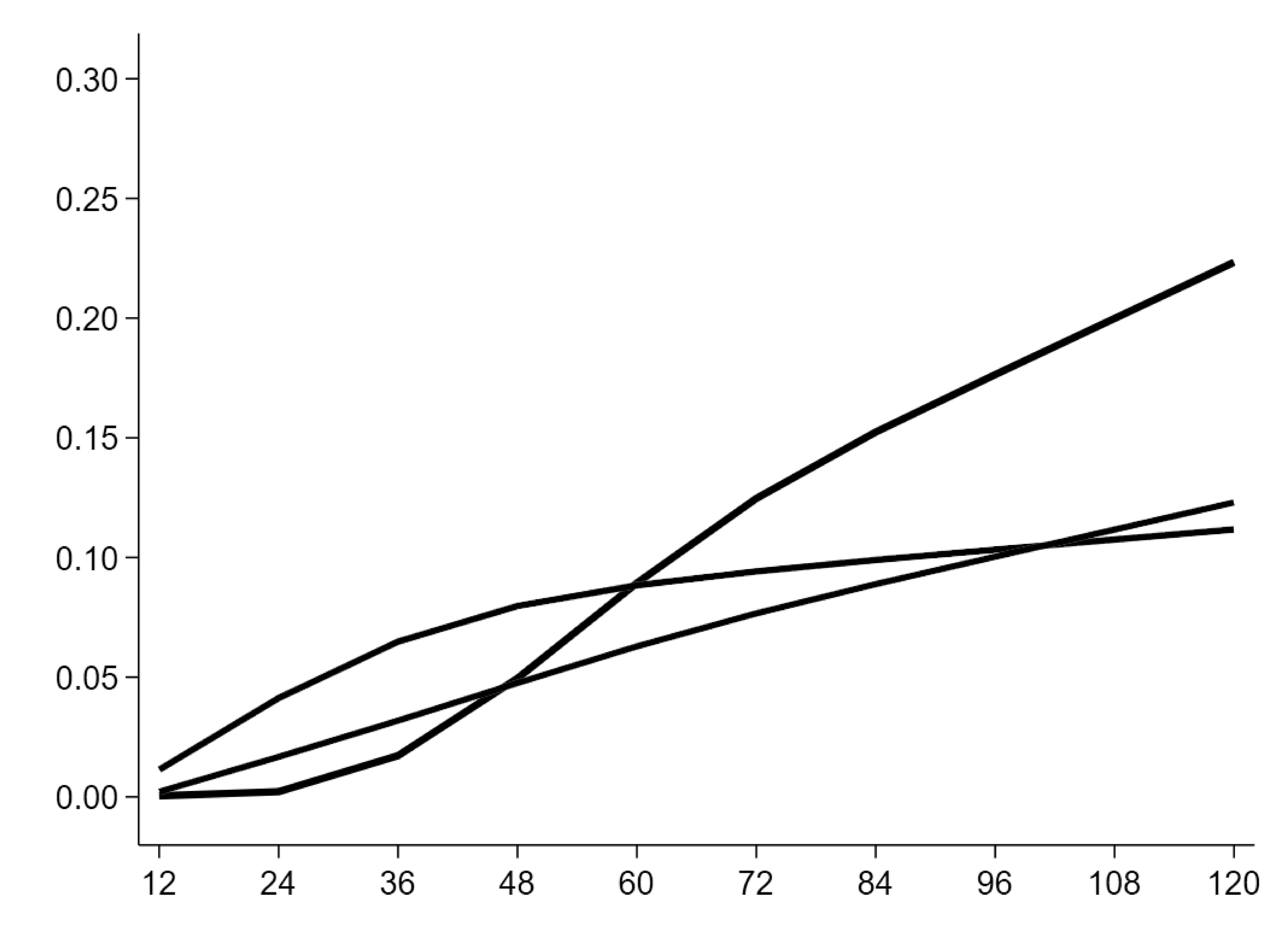

Supplement: Supplemental Material [file IANN_A_2521443_SM7117.zip › suppl_data/Supplementary Figure S2.tif]

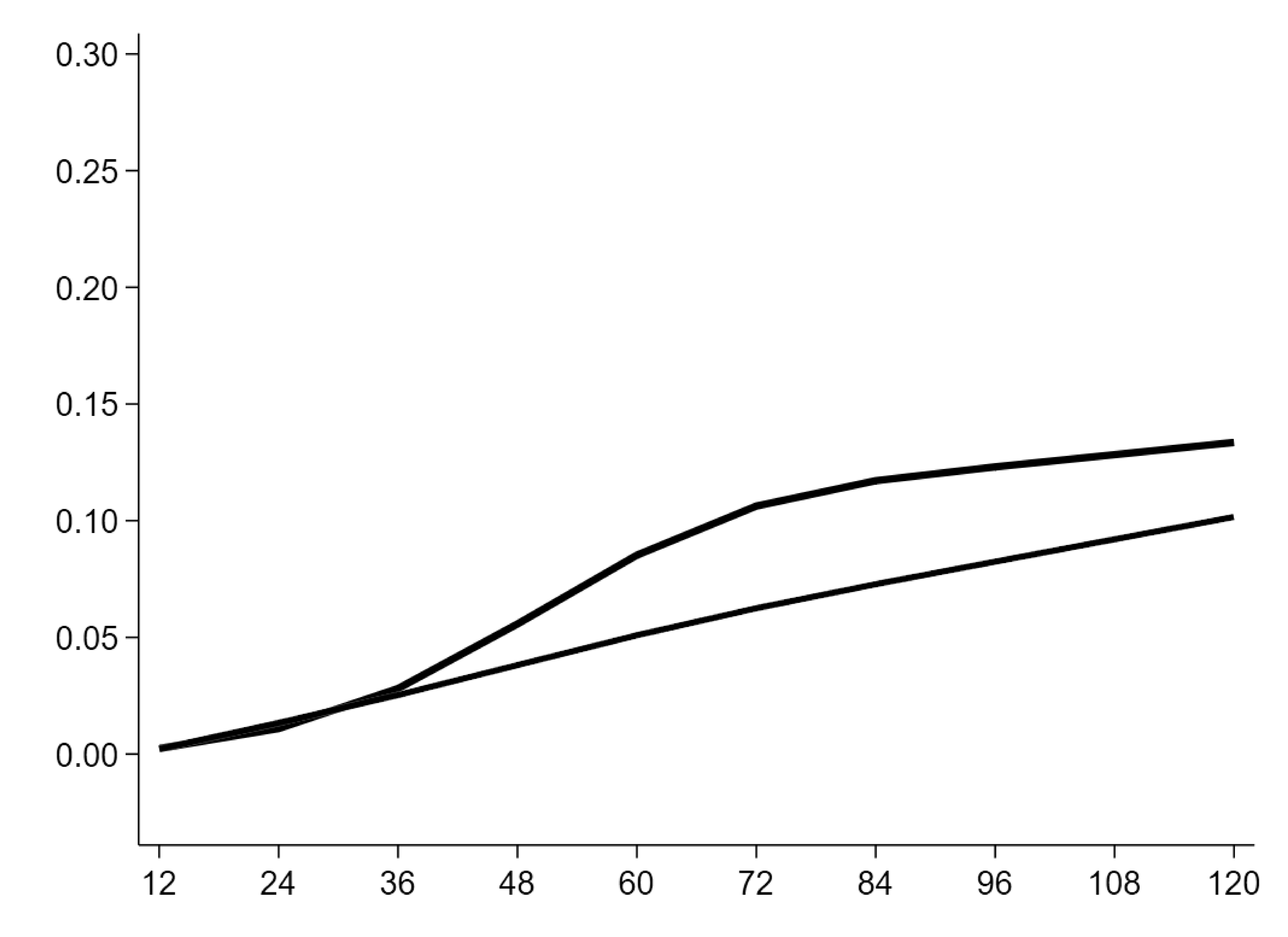

Supplement: Supplemental Material [file IANN_A_2521443_SM7117.zip › suppl_data/Supplementary Figure S3.tif]

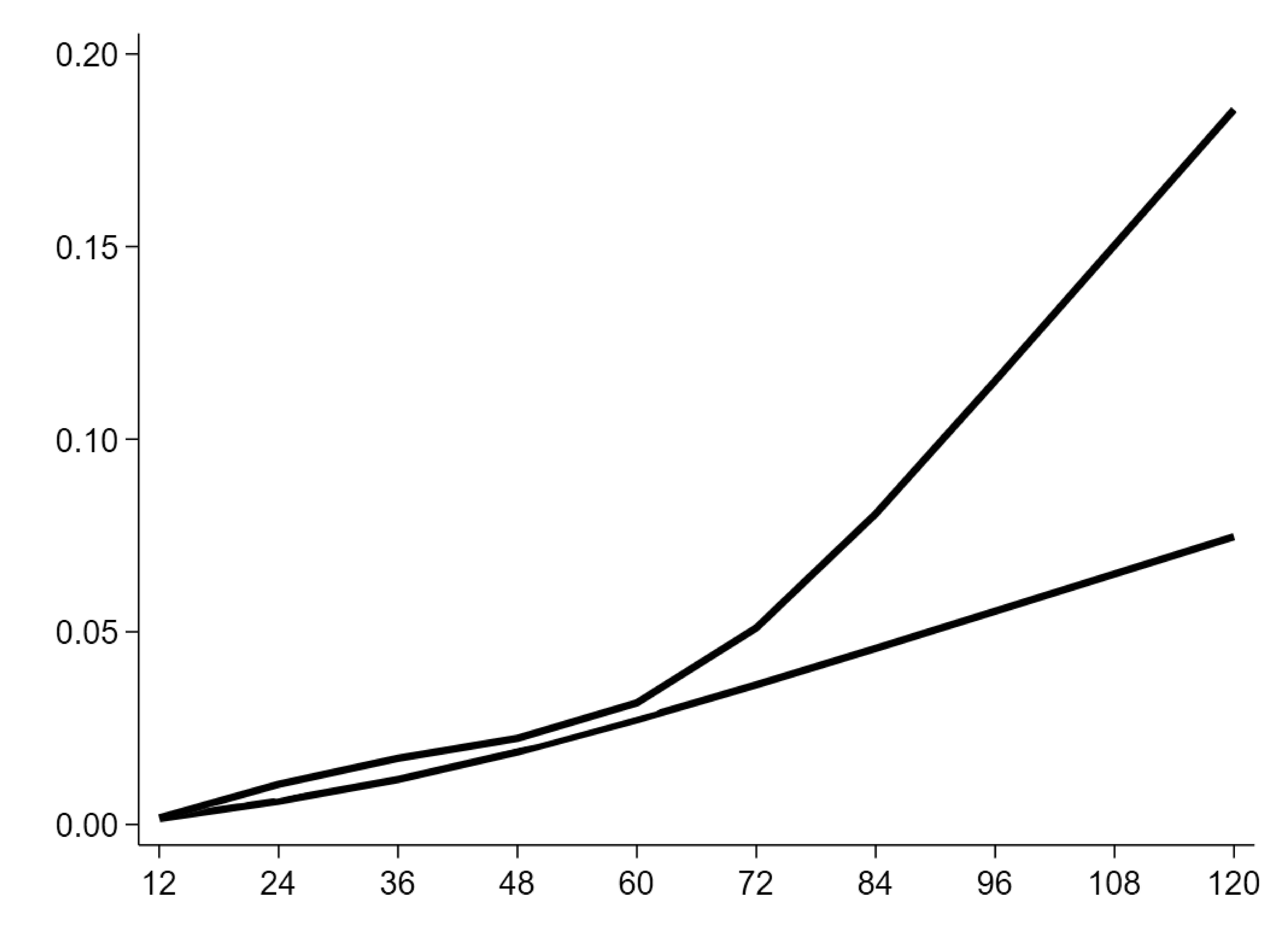

Supplement: Supplemental Material [file IANN_A_2521443_SM7117.zip › suppl_data/Supplementary Figure S4.tif]

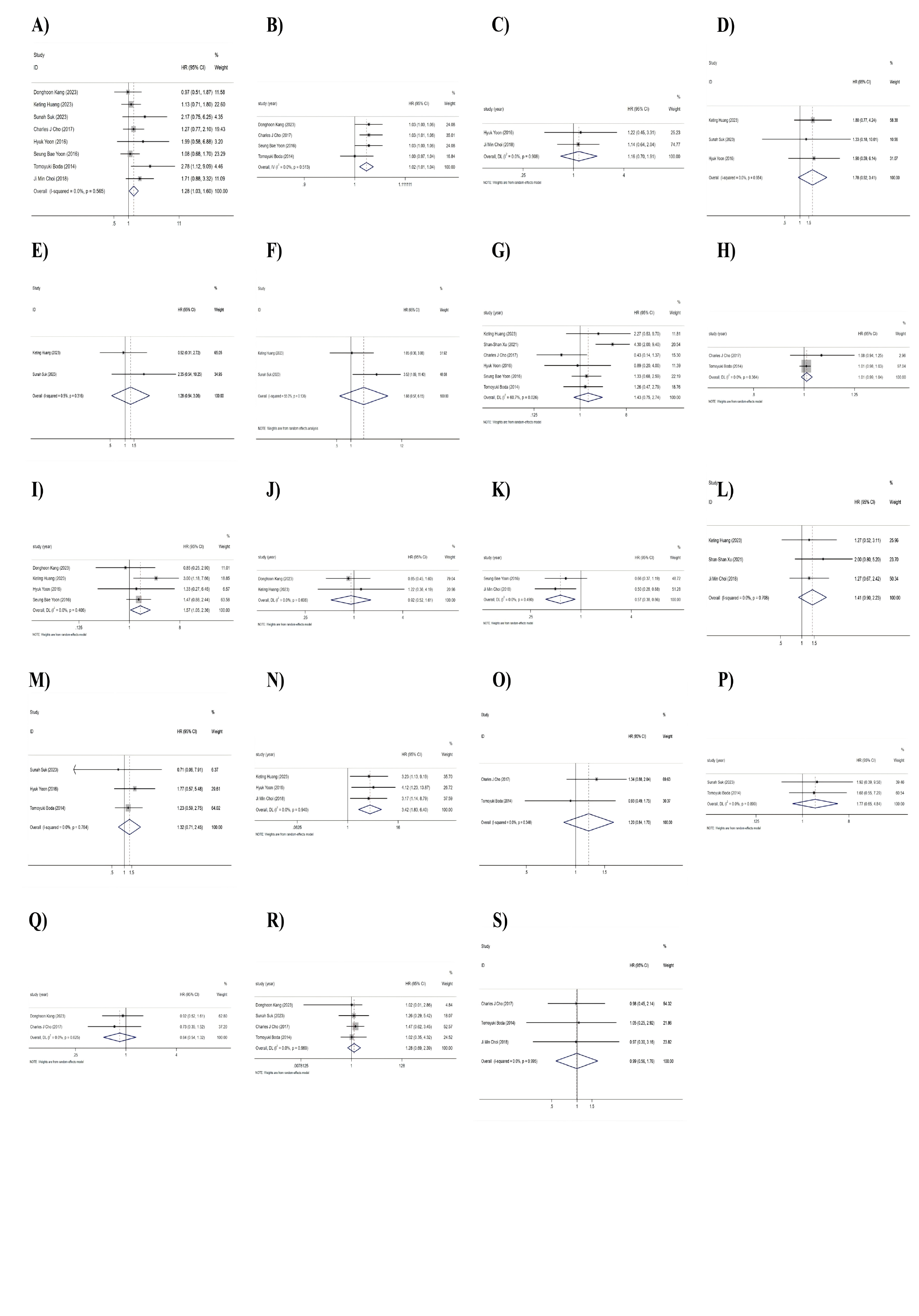

Supplement: Supplemental Material [file IANN_A_2521443_SM7117.zip › suppl_data/Supplementary Figure S5.tif]

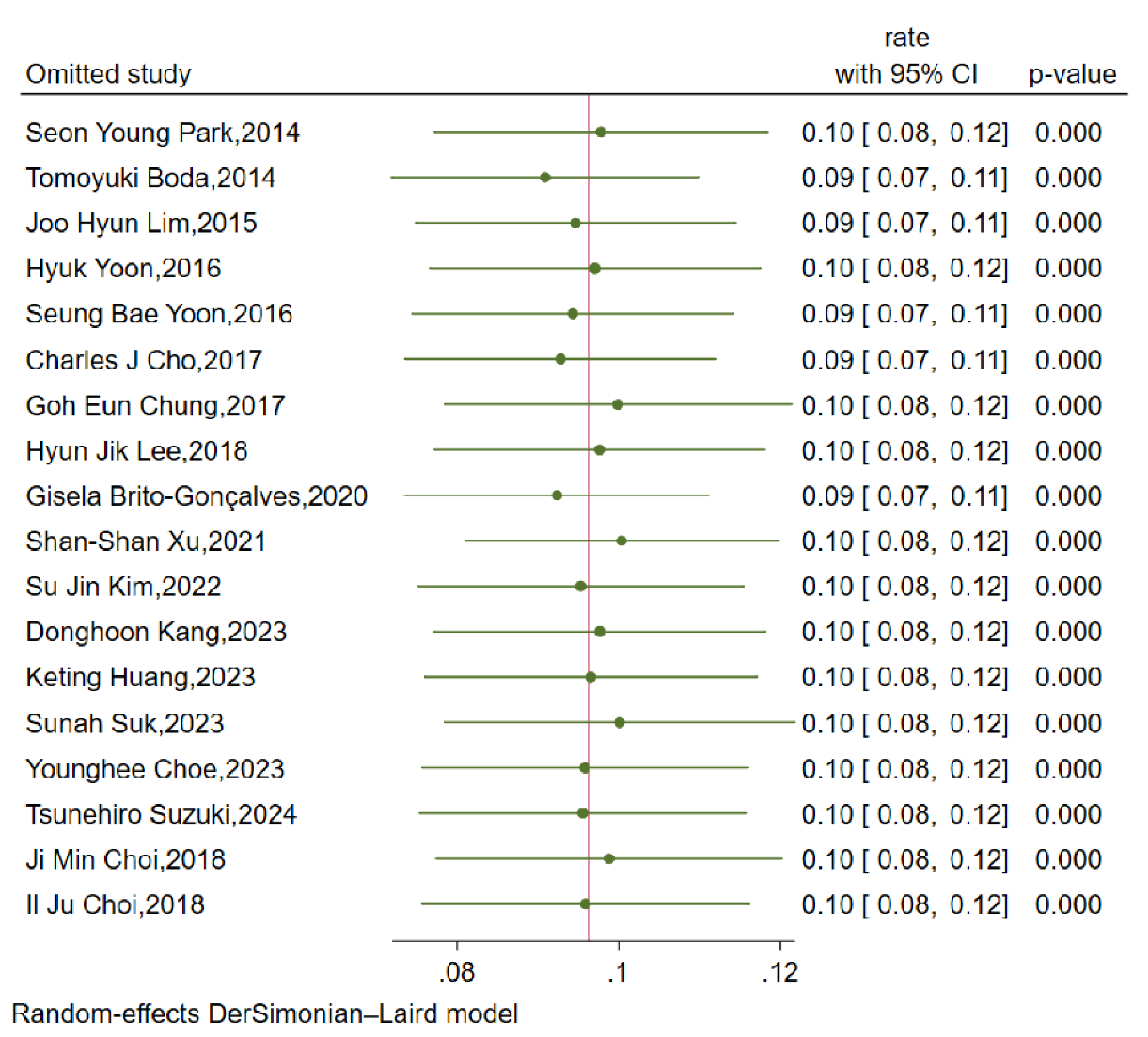

Supplement: Supplemental Material [file IANN_A_2521443_SM7117.zip › suppl_data/Supplementary Figure S6.tif]
